# Oral Levothyroxine for Myxedema Crisis: A Case Series from a Tertiary Referral Center

**DOI:** 10.1210/jendso/bvaf202

**Published:** 2025-12-13

**Authors:** Oriana Arias-Valderrama, Valentina Morales, Andrés Felipe Peña Arciniegas, Camila Pérez Tellez, Andrés Octavio García, Guillermo Édison Guzmán Gómez, Juan Lukas Ordóñez Giraldo

**Affiliations:** Facultad Salud, Departamento Medicina, Universidad Icesi, Cali 760032, Colombia; Fundación Valle del Lili, Centro de Investigaciones Clínicas, Cali 760032, Colombia; Facultad Salud, Departamento Medicina, Universidad Icesi, Cali 760032, Colombia; Facultad Salud, Departamento Medicina, Universidad Icesi, Cali 760032, Colombia; Facultad Salud, Departamento Medicina, Universidad Icesi, Cali 760032, Colombia; Departamento de Medicina Interna Endocrinología, Fundación Valle del Lili, Cali 760032, Colombia; Departamento de Medicina Interna Endocrinología, Fundación Valle del Lili, Cali 760032, Colombia; Fundación Valle del Lili, Centro de Investigaciones Clínicas, Cali 760032, Colombia

**Keywords:** myxedema, coma, endocrine emergency, severe hypothyroidism

## Abstract

**Introduction:**

Myxedema coma is the most severe form of decompensated hypothyroidism and represents a rare but life-threatening endocrine emergency. Standard treatment involves IV levothyroxine; however, access to this formulation is limited in many low-resource settings. This study aimed to describe the clinical characteristics, management, and outcomes of patients with myxedema coma treated with high-dose oral levothyroxine in a tertiary referral center in Colombia.

**Materials and Methods:**

We conducted an observational study of adult patients diagnosed with myxedema coma between January 2011 and December 2021 at Fundación Valle del Lili. Diagnosis was based on Popoveniuc criteria (>60 points) or presence of coma in the context of hypothyroidism. Data were collected from electronic medical records. All patients received oral levothyroxine, and clinical, laboratory, and outcome variables were analyzed.

**Results:**

Twelve patients were included (median age 66 years; 50% female). The most common precipitating factor was acute infection (41.6%). All patients received an oral loading dose of levothyroxine (median 500 µg), followed by high-dose maintenance therapy. Normalization of free T4 was observed in all patients by the fourth day. The intensive care unit admission rate was 100%, with a median stay of 11 days. Vasopressor support was required in 58.3%, and in-hospital mortality was 25%.

**Conclusion:**

High-dose oral levothyroxine was effective in achieving early biochemical recovery in patients with myxedema coma. In settings where IV formulations are unavailable, oral therapy represents a viable and potentially life-saving alternative.

Myxedema coma represents the most severe manifestation of decompensated hypothyroidism and is considered 1 of the endocrine emergencies with the highest morbidity and mortality worldwide. Its incidence ranges from 0.22 to 1 case per million inhabitants per year, with a higher prevalence in women over 60 years of age (female-to-male ratio of 8:1) [1-3].

Early diagnosis, prompt administration of thyroid hormones, and comprehensive supportive care are crucial to improving prognosis. According to the American Thyroid Association guidelines for the management of hypothyroidism, high-dose parenteral levothyroxine (LT4) replacement therapy is the treatment of choice for myxedema coma [3]. IV therapy is widely accepted and effective in managing hypothyroid crisis; however, IV LT4 and liothyronine are expensive, not readily accessible, and may have deleterious cardiovascular effects—particularly in patients with underlying coronary artery disease [4].

In resource-limited settings where IV LT4 is unavailable, enteral administration—via nasogastric tube or rectal route, depending on patient tolerance—is considered a viable alternative [5]. Nevertheless, due to the rarity of myxedema coma, there are no universally established protocols for the dosing and titration of enteral LT4. Furthermore, no randomized controlled trials or prospective observational studies have compared outcomes between IV and enteral administration. Current enteral treatment strategies are primarily based on expert opinion and isolated case reports.

In Colombia, IV LT4 is not available; thus, high doses of oral LT4 have been used for the management of this condition. Our institution follows this practice, and herein we present our clinical experience over the past few years.

## Materials and Methods

### Study Design

We conducted a retrospective observational study from January 2011 to December 2021, including patients diagnosed with myxedema who received treatment at our institution. Patients were identified through the hospital's electronic medical records using the International Classification of Diseases, Tenth Revision, codes E03.5 to E03.9. Relevant clinical data were extracted from the database, and individual medical records were reviewed according to the study objectives.

### Study Setting

This study was carried out at Fundación Valle del Lili, a tertiary referral center for critical care located in southwestern Colombia. The institution is equipped with advanced diagnostic tools and specialized personnel and provides access to high-quality treatments and medications.

### Variables and Data Collection

We collected demographic data (eg, age), clinical variables (eg, length of hospital stay, comorbidities, clinical presentation, and diagnostic parameters related to hypothyroidism), and laboratory findings including TSH and free T4 levels measured by electrochemiluminescence. Notably, antithyroid antibodies (thyroid peroxidase, triglyceride) were not measured, as this is not routinely performed in Colombia for such cases. Key clinical outcomes, including occurrence of infections, shock, and in-hospital mortality, were also recorded.

### Inclusion and Exclusion Criteria

We included adult patients (≥18 years) diagnosed with myxedema coma who received treatment in the hospital and met Popoveniuc criteria with a score >60 or presented with coma secondary to hypothyroidism. Pregnant patients were excluded from the analysis.

### Statistical Analysis

Descriptive statistics were used to summarize the clinical characteristics of the study population. Categorical variables were presented as frequencies and percentages. Continuous variables were presented as measures of central tendency and dispersion according to their distribution. All analyses were performed using RStudio, version 2022.07.2.

## Results

A total of 12 patients were included, with a median age of 66 years [interquartile range (IQR): 59-74]. Sex distribution was equal, with 6 male (50%) and 6 female (50%) patients. A history of hypothyroidism was present in 5 patients (41.6%). Only 1 patient (8.3%) had a history of heart failure. All cases were classified as hypothyroidism of unclear etiology. Hypothermia was documented in 6 patients (50%). Although infectious processes were systematically ruled out in all 12 cases upon admission, a single patient (8.3%) was febrile, which was attributed to a severe *Clostridioides difficile* infection. Altered mental status on admission was observed in 10 patients (83.2%). The most frequently identified cause of myxedema coma was acute infection (5/12, 41.6%), followed by unknown causes (5/12, 41.6%), and failure to adhere to thyroid hormone therapy (2/12, 16.6%) (Table 1).

**Table 1.** Characteristics of patients

Characteristic	n = 12
Age, median (IQR)	66 (59-74)
Sex, n (%)	
Male	6 (50)
Female	6 (50)
History of hypothyroidism, n (%)	5 (41.6)
History of arterial hypertension, n (%)	11 (91.6)
History of heart failure, n (%)	1 (8.3)
Hypothyroidism without clear cause, n (%)	12 (100)
History of treatment with levothyroxine, n (%)	5 (41.6)
Heart rate, median (IQR)	60 (54-75)
Heart rate < 60 bpm, n (%)	11 (91.7)
Heart rate >100 bpm, n (%)	1 (8.3)
Hypothermia, n (%)	6 (50)
Fever, n (%)	1 (8.3)
Alteration of consciousness on entrance to hospital, n (%)	
Somnolence	8 (66.6)
Stupor	2 (16.6)
Cause of myxedema coma, n (%)	
Acute infection	5 (41.7)
Unknown	5 (41.7)
Failure of adherence to treatment	2 (16.6)

Abbreviation: IQR, interquartile range.

Although the dosing strategy appeared standardized, it is important to note that the initial LT4 doses were ultimately determined by the attending physicians, guided by their clinical judgment and experience. In the absence of robust evidence or formal guidelines for oral LT4 use in myxedema coma, treatment decisions were individualized based on patient stability, comorbidities, and physician discretion. Nonetheless, all patients in this series received the same initial oral LT4 loading dose of 500 µg, followed by 200 µg/day for the subsequent 3 days. Maintenance therapy was then continued at a weight-based dose of 1.7 µg/kg/day. Empiric corticosteroid therapy with IV hydrocortisone was administered in 10 patients, with a median duration of 7 days (Table 2). Free T4 levels normalized in all patients by the fourth day of treatment, indicating a prompt and effective biochemical response to high-dose oral LT4 (Table 3).

**Table 2.** Treatment

Characteristic	n = 12
First dose of levothyroxine, loading dose (µg), median (IQR)^*a*^	500 (200-500)
Maintenance dose of levothyroxine (µg/day), median (IQR)^*a*^	200 (100-300)
Days of levothyroxine, median (range)	3 (3-270)
Corticosteroids (hydrocortisone), n (%)	10 (83.3)

Abbreviation: IQR, interquartile range.

^a^High dose defined as ≥200 µg/day (initial loading dose: 500 µg; maintenance high do**s**e: 200 µg/day.

**Table 3.** Blood level hormones

Characteristic	Day 1	Day 2	Day 3	Day 4	Day 5
TSH, uU/mL, median (IQR)	157 (105-208)	134 (59.4-226.3)	30.0 (15.6-50.8)	8.97 (1.4-98.6)	10.4 (4.7-64.1)
Free T4, ng/dL, median (IQR)	0.33 (0.18-0.55)	0.7 (0.47-0.72)	1.04 (0.8-1.13)	1.21 (1.02-1.32)	1.32 (1.2-1.38)

Abbreviation: IQR, interquartile range.

All patients (12/12, 100%) required admission to the intensive care unit. The median intensive care unit length of stay was 11 days (IQR: 5-27). Airway support was required in 6 patients (50%), and vasopressor therapy was administered to 7 patients (58.3%). The median APACHE II score at admission was 15.5 (IQR: 12-20), and the median SOFA score was 5.5 (IQR: 4-7). The overall in-hospital mortality rate was 25% (3/12) (Table 4). The causes of death for these 3 patients were as follows: patient 1 died from the progression of respiratory deterioration associated with airway obstruction secondary to a previously documented metastatic tracheal lesion; patient 2 died of septic shock of abdominal origin secondary to complicated diverticular disease; and patient 3 died from a severe *Clostridioides difficile* infection with therapeutic failure after 10 days of treatment.

**Table 4.** Outcomes

Characteristic	n = 12
ICU, median (IQR)	12 (100)
Days of stay in ICU, median (IQR)	11 (5-27)
Days following the initiation of LT4, median (IQR)	3 (3-270)
Airway support, n (%)	6 (50)
Vasopressors, n (%)	7 (58.3)
Shock, n (%)	
Septic shock	3 (25)
Obstructive shock	1 (8.3)
Cardiogenic shock	1 (8.3)
APACHE score, median (IQR)	15.5 (12-20)
SOFA score, n (%)	5.5 (4.7)
Days of mechanical ventilation, n (%)	1 (0.7)
Infection, n (%)	7 (58.3)
Death, n (%)	3 (25)

Abbreviations: ICU, intensive care unit; IQR, interquartile range; LT4, levothyroxine.

## Discussion

In this case series, we describe our clinical experience managing myxedema coma with high-dose oral LT4 in a tertiary care center where the IV formulation was unavailable. Our findings suggest that high-dose oral LT4 was associated with early biochemical recovery—specifically, normalization of free T4 levels by day 4 in all patients—and with favorable clinical outcomes.

Predicting outcomes in myxedema coma remains challenging due to the rarity of the condition, with reported mortality rates ranging from 25% to 80%. This wide range may be attributable to inconsistencies in determining whether mortality was directly caused by the complications of severe hypothyroidism or by unrelated, preexisting comorbidities in these critically ill patients. Factors associated with increased mortality include advanced age, low Glasgow Coma Scale scores, and elevated APACHE II and SOFA scores. Nevertheless, neither the underlying etiology of hypothyroidism (primary vs secondary) nor the route of LT4 administration (oral vs IV) appears to significantly influence mortality [1, 6-9]. In our case series, the in-hospital mortality rate was 25%, a figure that falls at the lower end of the wide spectrum reported in the literature. These findings suggest that, when appropriately dosed and supported with intensive care, oral LT4 may serve as an effective alternative in resource-limited settings.

This study has several limitations inherent to its observational design and the rarity of myxedema coma. First, the small sample size (n = 12) reflects the low incidence of this condition, which complicates the generalizability of our findings. However, our cohort represents 1 of the largest single-center case series reported in the literature to date, underscoring the challenges of conducting prospective studies in this clinical context. Future multicenter collaborations are warranted to validate these results in larger populations. Second, the absence of a control group treated with IV LT4 limits direct comparisons between administration routes. To provide context for our findings, we note that our observed mortality rate of 25% is consistent with the lower end of the wide mortality range (25-80%) documented in previous reports, which largely consist of larger database analyses or multicenter cohorts [1, 6-9]. We acknowledge that a direct statistical comparison is not feasible due to fundamental differences in study design, scale, and follow-up duration between our single-center series and these larger studies. Notably, in Colombia—where IV LT4 is unavailable—our institution's standardized high-dose oral protocol emerged as the sole viable option for these critically ill patients. Finally, postdischarge follow-up data (eg, recurrence rates, long-term functional outcomes) were not systematically collected, precluding assessment of sustained recovery. Prospective studies should incorporate extended monitoring periods to address this gap.

The severity of this medical condition, reflected in its high morbidity and mortality, underscores the urgent need for thyroid hormone replacement—regardless of the route of administration (oral or parenteral)—to rapidly restore thyroid hormone levels to normal. Notably, publications involving patients treated with oral LT4 have shown biochemical and clinical outcomes comparable to those observed with IV therapy [6, 10]. In patients with myxedema coma treated with LT4, oral doses were typically higher due to the reduced bioavailability of the oral formulation. This is likely related to the slower and more gradual absorption via the gastrointestinal tract.

The American Thyroid Association recommends an IV LT4 loading dose of 200 to 400 µg. However, several case series have reported the use of higher doses. The loading dose should be followed by daily doses of 150 to 200 µg for the next 2 days and subsequently adjusted to 1 to 1.6 µg/kg/day until discharge. Dosing should be individualized based on the presence of comorbidities, with lower doses recommended for smaller patients, elderly individuals, and those with a history of coronary artery disease or arrhythmias [6, 11, 12]. In this regard, Rajendran et al proposed that oral LT4 dose be stratified into 3 clinical categories: (1) patients without coronary artery disease: loading dose of 500 µg, followed by 200 µg for 2 days, then 150 µg for 2 days, and 1.6 to 2.0 µg/kg/day at discharge; (2) patients with coronary artery disease and preserved left ventricular ejection fraction: loading dose of 300 to 400 µg, followed by 200 µg for 2 days, then 150 µg for 2 days, and 1.6 to 2.0 µg/kg/day at discharge; and (3) patients with coronary artery disease and reduced left ventricular ejection fraction: loading dose of 250 to 300 µg, followed by 150 µg for 2 days, then 100 µg for 2 days, and 1.0 to 1.6 µg/kg/day at discharge [6]. This approach resulted in 1 of the lowest mortality rates reported to date. In our experience, an oral loading dose of 500 µg followed by 200 µg/day for 3 days, then transitioned to a maintenance dose of 1.7 µg/kg/day, yielded favorable outcomes with low mortality. Given the absence of clinical trials, it is not possible to determine whether more individualized dosing would have led to further improvements in mortality among our patients.

## Conclusion

In healthcare environments without access to IV LT4, oral administration offers a practical substitute. Despite this recommendation, there are no clinical trials comparing the effectiveness of IV vs oral administration. Consequently, no standardized protocols with high-quality evidence are currently available, and their use has been primarily supported by small observational studies. This study contributes to the growing body of evidence supporting oral LT4 as a safe and effective alternative for the treatment of a rare but highly lethal condition.

## Data Availability

All data generated or analyzed during this study are included in this published article.
